# Editorial: Combating Antimicrobial Resistance - A One Health Approach

**DOI:** 10.3389/fcimb.2019.00458

**Published:** 2020-01-22

**Authors:** Ghassan M. Matar, Antoine Andremont, Wael Bazzi

**Affiliations:** ^1^Department of Experimental Pathology, Immunology and Microbiology, Center for Infectious Diseases Research (CIDR) and WHO Collaborating Center for Reference and Research on Bacterial Pathogens, Faculty of Medicine, American University of Beirut, Beirut, Lebanon; ^2^Paris Diderot University Medical School, Paris, France

**Keywords:** resistance mechanisms, combination therapy, novel antibiotics, natural products, mode of action, therapeutics, antimicrobials, inhibitors

Antimicrobial resistance (AMR) is a life threatening and a very serious global health problem. There is an increasing alarming concern regarding the emergence of multi-drug resistant (MDR) superbugs. Infections emerging from such pathogens are in most cases unresponsive to treatment with few, if any, antimicrobial agents currently available and effective. This issue is causing the development of two eras, a pre-antimicrobial agent era and raises concerns regarding a post-antimicrobial agent era, where the last resort antimicrobial agents are not potent to treat infections caused by Gram-positive and Gram-negative MDR strains.

Given the complexity of the AMR challenge at the level of human and animal health, in addition to the impact on the environment, it seems very important to stress and emphasize the role of a “One Health” approach in tackling the problem of AMR. Therefore, unifying efforts to combat and overcome this alarming issue requires a many-sided approach. The key points of this mission are mainly highlighted in three major tasks, understanding the bacterial resistance mechanisms, in addition to the utilization of combinatorial therapeutic approaches for potential clinical options, and the discovery of novel antimicrobial agents and/or targets.

The Research Topic entitled: “Combating Antimicrobial Resistance—A One Health Approach” harbors 25 published manuscripts that include 197 authors, scientists and researchers from worldwide renowned institutions and research centers. These manuscripts mainly focus on understanding the mechanisms of resistance in Gram-positive and Gram-negative MDR strains, address combination antimicrobial therapy as a potential treatment against Gram-positive and Gram-negative bacilli, and the discovery of novel antimicrobial agents with potential novel targets or new mode of actions.

To understand the mechanisms of resistance in Gram-positive and Gram-negative MDR strains, the Research Topic consists of 13 outstanding manuscripts, reviews, and original research articles that aim to elucidate novel mechanisms of resistance in different MDR strains and widen our knowledge to further understand how Gram-positive and Gram-negative bacteria combat antimicrobial agents.

With respect to Gram-positive bacteria, molecular epidemiological studies in *Streptococcus pneumoniae* from children with pneumonia in “Shanghai, China” provided the microbiology community with precise prevalence of serotype modifications and diversity in the emergence of MDR international clones, where the most common serotypes documented are 19F, 6A, 19A, 23F, 14, and 6B (Zhao et al.). Interestingly, the epidemiological study in *S. pneumoniae* correlates with the emerging crisis of AMR in China. This crisis is worsening and has become a major public and global safety problem, causing serious danger to humans and animal health and to the environment. This is due to the fact that the emergence of bacterial resistance is much faster as compared to the discovery of new antimicrobial agents, which will lead humans to enter the “post-antimicrobial era.” This makes China one of the most nations at risk of augmented AMR (Qu et al.). Furthermore, poultry surveillance studies in the UK have indicated the presence of livestock-associated methicillin-resistant *Staphylococcus aureus* (LA-MRSA) which suggests an augmenting problem in the zoonotic transmission of LA-MRSA in Europe (Anjum et al.). In addition, AMR poultry studies in the US revealed the possible propagation of resistance genes to the soil through poultry waste. This highlights the high need to understand the migration and the spread of AMR genes in the environment and in particular the ones in direct relevance to the health of humans and animals using the One Health Approach (Yang et al.).

In relation to Gram-negative bacteria, in the past decade the Middle East has become a reservoir for extended-spectrum cephalosporin and carbapenem resistant Gram-negative bacilli (GNB) in hospitals and to a lower level in the environment. Carbapenemases producers are dominating in hospitals while, extended spectrum beta lactamases (ESBL) and colistin resistance are becoming an alarming concern in animals. This is mainly due to the abusive use of colistin in veterinary medicine, where minimal information is available regarding the level of antimicrobial agents' consumption in the whole community and in farms (Dandachi et al.).

Integrative and conjugative elements (ICEs) are self-transmissible mobile genetic elements that provide the bacteria with all necessary tools to acquire complicated and complex traits through horizontal gene transfer and encode resistance to antimicrobial agents and heavy metals in parallel through a process known as “co-selection.” Assessing the emergence of multidrug- and toxin-resistant bacteria via ICEs in *Vibrio parahaemolyticus*, isolated from aquaculture shrimp in China revealed that ICEs are not the major transmission facilitators of resistance to antimicrobial agents or heavy metals in Asia (He et al.). In addition to ICE, “Heteroresistance” is one of the mostly investigated aspects that are a characteristic of most MDR pathogens. One important issue is the absence of a universal guideline that clinicians could apply to treat heteroresistant *Helicobacter pylori* in clinical settings. Therefore, increased knowledge of gastroenterologists about the presence of the heteroresistance phenomenon is highly essential. This should be accompanied with accurate breakpoint measurements in order to have a firm statement of heteroresistance among various *H. pylori* isolates (Rizvanov et al.).

To further understand the resistance mechanisms to antimicrobial agents in Gram-negative MDR strains, several studies documented the molecular and physiological characterization of fluoroquinolone resistance and the mechanisms of adaptive resistance to aminoglycosides in *Salmonella enteritidis* and *Escherichia coli*, respectively. In *S. enteritidis*, two high-frequency mutations can take place in the *gyrA* gene, leading to amino acid substitutions S83Y, S83F, and D87G. These mutations induce augmented resistance to fluoroquinolones as they upregulate the expression levels of the efflux pump associated genes *ramA, acrB*, and *tolC*, and downregulate the relative expression levels of the porin gene *ompF* and thus, lead to high levels of AMR (Vidovic et al.). In *E. coli, yhjX* encodes a putative transporter, known to be the only target of the YpdA/YpdB two-component system, which in turn is mainly induced by pyruvate availability. *yhjX* deletion improves the growth of *E. coli* in a medium containing sub-inhibitory concentrations of gentamicin. With time, *yhjX* mediates the adaptive resistance to aminoglycosides (Zhou et al.).

Overcoming AMR, inhibiting biofilm formation, and the inhibition of diarrhea and death caused by pathogenic *E. coli* in newborn piglets were documented in two research articles. In the former, the spore forming, rod-shaped, Gram-positive *Bacillus subtilis* was used as it is known to be a safe and a reliable probiotic in humans and animals. The isolation of *B. subtilis*, named WS-1, from healthy pigs inhibits the growth of pathogenic *E. coli in vitro* and protects the small intestine from injuries caused by an *E. coli* infection (Du et al.). In the latter study, seven gene-specific knockouts were produced by recombination in ocular *E. coli*. The mutations targeted three transmembrane genes *ytfR, mdtO*, and *tolA, ryfA* coding for non-coding RNA and three metabolic genes *mhpA, mhpB*, and *bdcR*. Interestingly, biofilm synthesis was inhibited in five mutants (Δ*bdcR*, Δ*mhpA*, Δ*mhpB*, Δ*ryfA*, and Δ*tolA*) with no impact on their susceptibility profiles against a panel of antimicrobial agents such as ceftazidime, cefuroxime, ciprofloxacin, gentamicin, cefotaxime, sulfamethoxazole, and imipenem. Indeed, this was the first study to demonstrate the role of metabolic genes in biofilm formation (Ranjith et al.).

Although AMR genes can remain for decades in the environment even in an antimicrobial-deprived condition, reducing the use of antimicrobials is a key step to decrease AMR in livestock. Fecal complex microbial communities can out-compete bacteria harboring AMR genes. Interestingly, it was shown that changing the microbial surrounding can inhibit the transmission of AMR genes from one generation to another. For example, the proportion of resistant *Enterobacteria* declined from 93 to 9% accompanied with reduced prevalence of eight AMR genes upon testing different fecal complex microbial communities in rabbits (Achard et al.). In a similar study that focused on the effect of perinatal tulathromycin (TUL) metaphylaxis on the development of fecal microbiota and its correlation with AMR in pre-weaned piglets, it was shown that a total of 127 AMR genes linked to 19 different classes of antimicrobial agents are present. However, these results highlighted that perinatal TUL metaphylaxis has no essential impacts on fecal microbiota structure and the prevalence of AMR genes (Zeineldin et al.). These two studies suggest that different fecal microbial communities impact sensitivity to different classes of antimicrobial agents in addition to the abundance of AMR genes.

In the context of applying a combinatorial therapeutic approach for potential treatment options against MDR Gram-positive and Gram-negative bacteria and the discovery of novel antimicrobial agents and/or targets, the Research Topic consists of 12 manuscripts from well-recognized research institutes and universities.

With respect to Gram-positive bacteria, *in vitro, ex vivo*, and *in vivo* studies to assess the efficacy of the antimicrobial peptide DPK-060 revealed promising results in treating *S. aureus* topical infections. DPK-60 significantly decreased bacterial counts and bacterial survival rates, highlighting DPK-60 as a potential safe drug pending further clinical trials (Håkansson et al.). In addition to DPK-60, the synthetic retinoid compound CD437 was shown to have strong bactericidal effects on *Enterococcus faecalis*, anti-biofilm effects on *Staphylococcus*, and reduced biofilm synthesis in *Pseudomonas aeruginosa*. This study was performed in China and interestingly, it highlighted synergistic effects when CD437 was combined with gentamicin (Tan et al.). This proves the effectiveness of combination therapies to further enhance the potency of specific drugs. In a similar study, Balsacone C was shown to inhibit MRSA isolates without inducing resistance. Balsacone C is a new dihydrochalcone isolated from *Populus balsamifera* that was shown to have strong inhibitory effects on *S. aureus*. Upon *in vitro* assessment, Balsacone C induced sensitivity on a panel of antimicrobial agents such as penicillin, amoxicillin/clavulanic acid, ciprofloxacin, moxifloxacin, levofloxacin, clindamycin, erythromycin, and cefoxitin. In addition, microscopic analysis revealed that Balsacone C can induce changes in the bacterial cell membrane and thus, demonstrating the mode of action of Balsacone C (Côté et al.).

In relation to Gram-negative bacteria, a study from China highlighted the usage of anti-outer membrane vesicle antibodies to increase antimicrobial sensitivity of Pan-Drug Resistant (PDR) *Acinetobacter baumannii*. Mice were immunized with *A. baumannii* outer membrane vesicles (AbOMVs) to produce high IgG levels. The produced antibodies were then injected in mice infected with *A. baumannii*. Interestingly, this process enhanced the susceptibility to quinolone antimicrobial agents, improved the survival rate of mice, and decreased bacterial load in organs. This sheds light onto a novel approach to treat PDR *A. baumannii* (Huang et al.). In a different study performed in Australia, the antimicrobial activity of Robenidine alone or in combination with ethylenediaminetetraacetic acid (EDTA) or polymyxin B nonapeptide (PMBN) against Gram-negative pathogens was assessed *in vitro* using the Broth Microdilution (BMD) assay and combinatorial tests such as the checkerboard method. Interestingly, the assessment of robenidine alone revealed bactericidal effects against *A. baumannii* and *Acinetobacter calcoaceticus*. In combination with either EDTA or PMBN, robenidine showed antimicrobial activity against *E. coli, Klebsiella pneumoniae*, and *P. aeruginosa* with no anti-biofilm effects. This study reveals for the first time the impact of the combination of robenidine with EDTA or PMBN for potential clinical application (Khazandi et al.).

Bacterial persister cells are unique cells that exhibit phenotypic modifications and are known to tolerate the effect of antimicrobial agents in a transient manner and play important roles in the emergence of AMR and chronic infections. The formation of persister cells is not well-established. In a study from China, it was shown that hypoionic shock facilitates aminoglycoside killing of both nutrient shift- and starvation-induced bacterial persister cells in *E. coli*, but not *S. aureus*. Hypoionic shock facilitates aminoglycoside uptake and this study sheds light on the importance of further investigating persister cells eradication and aminoglycoside use (Chen et al.).

*Candia albicans* resistance to antifungal agents has been well-documented in the past years, which raised concerns and challenges in clinical therapeutic treatments. It was shown that N-butylphthalide extracted from *Apium graveolens* has potential antifungal activity and anti-biofilm effects against *C. albicans*. Interestingly, upon the combination of N-butylphthalide with fluconazole, further synergistic effects were observed. Additional experiments from this study reveal that the documented synergistic effects are due to increased cell uptake and decreased efflux pumping (Gong et al.).

Carbapenems are considered among the last-resort antimicrobial agents; however Carbapenem-resistant Gram-negative bacteria became major concerns in hospitals. β-Lactamases encoding genes such *KPC, NDM*, and *OXA* were shown to encode for carbapenem resistance as well. A new therapeutic strategy to combat Carbapenem-resistant Gram-negative bacteria involves the usage of carbapenem/β-lactamase inhibitor (βLI) combinations. Combining meropenem, imipenem, and ertapenem with the corresponding βLIs restored *A. baumannii*'s sensitivity *in vitro* and increased survival rates *in vivo* when meropenem was combined with Avibactam or Relebactam. This study highlights carbapenem/βLI combinations as a new option for antimicrobial combination therapies (El Hafi et al.). In a similar study, ceftazidime/avibactam enhanced the antibacterial potency of Polymyxin B against Polymyxin B heteroresistant KPC-2-Producing *K. pneumoniae* and inhibited the production of resistant bacteria *in vitro*. This study also suggested that the combination of polymyxin B and the ceftazidime/avibactam could be considered for treating heteroresistant bacteria (Ma et al.).

With respect to virulence in *K. pneumoniae*, the World Health Organization (WHO) has listed this bacterium as one of the major pathogens in immediate need for novel antimicrobial agents due to the wide spectrum of AMR. This was mainly highlighted in a review that focused on the challenges and concerns in establishing a universal *Klebsiella* vaccine and a novel drug due to the high frequency of mutations this pathogen acquires. This is represented via the various modifications *Klebsiella* strains can adopt at the level of the extracellular polysaccharides, such as lipopolysaccharides, capsular polysaccharides, and exopolysaccharides (Patro and Rathinavelan). In a different study focusing on MDR *K. pneumoniae* resistant to sulfonamides and β-lactams, the potency of commercially and clinically available sulfonamides animal feeds were examined to select for a model strain upon measuring the recovery rate in the presence of AMR markers *sul1* (sulfonamide) and *blaKPC-*3 (meropenem). This research study highlights that in certain conditions the use of non-essential antimicrobial agents can result in “co-selection” mechanisms and the emergence of resistance to essential antimicrobial agents, which might have direct consequences on the human health on the long-term (Brown et al.).

To predict clinical efficacy of novel antimicrobial agents, the VetCAST (the veterinary subcommittee of EUCAST) presents a semi-mechanistic time-kill curve (TKC) assay to assess drug potencies. For example, florfenicol, a long acting veterinary antimicrobial agent used against calf pneumonia organisms (*Pasteurella multocida* and *Mannheimia haemolytica*) was assessed in a collaborative study between France and US. It was shown that the semi-mechanistic approach provides precise estimation of bacterial growth, counts, and pharmacodymanic variables. Such studies are in high need nowadays in order to check whether novel antimicrobial agents are of significant impact to combat AMR (Pelligand et al.).

In conclusion, the published manuscripts included in this Research Topic help shed light on the growing problems of AMR, bacterial pathogenesis and virulence, in addition to understanding bacterial resistance, the utilization of combination therapy for potential therapeutic options, and the discovery of novel antimicrobial agents and/or targets in Gram-positive and Gram-negative MDR strains.

## Author Contributions

GM drafted and edited the manuscript. AA edited the manuscript. WB assembled all needed information and contributed to drafting the manuscript.

### Conflict of Interest

The authors declare that the research was conducted in the absence of any commercial or financial relationships that could be construed as a potential conflict of interest.

